# Identification, Characterization, and Optimization of Integrin α_v_β_6_-Targeting Peptides from a One-Bead One-Compound (OBOC) Library: Towards the Development of Positron Emission Tomography (PET) Imaging Agents

**DOI:** 10.3390/molecules24020309

**Published:** 2019-01-16

**Authors:** Yng (Sarah) C. Tang, Ryan A. Davis, Tanushree Ganguly, Julie L. Sutcliffe

**Affiliations:** 1 Department of Internal Medicine, Division of Hematology/Oncology, University of California, Davis and Sacramento, CA 95817, USA; yngtang@ucdavis.edu (Y.C.T.); tganguly@ucdavis.edu (T.G.); 2 Department of Biomedical Engineering, University of California, Davis, CA 95616, USA; rydavis@ucdavis.edu; 3 Center for Molecular and Genomic Imaging, University of California, Davis, CA 95616, USA; 4 Radiochemistry Research and Training Facility, University of California, Davis, Sacramento, CA 95817, USA

**Keywords:** one-bead one-compound (OBOC), integrin α_v_β_6_, library screening, positron emission tomography (PET), Fluorine-18, solid-phase radiolabeling, affinity, selectivity

## Abstract

The current translation of peptides identified through the one-bead one-compound (OBOC) technology into positron emission tomography (PET) imaging agents is a slow process, with a major delay between ligand identification and subsequent lead optimization. This work aims to streamline the development process of ^18^F-peptide based PET imaging agents to target the integrin α_v_β_6_. By directly identify α_v_β_6_–targeting peptides from a 9-mer 4-fluorobenzoyl peptide library using the on-bead two-color (OBTC) cell-screening assay, a total of 185 peptide beads were identified and 5 beads sequenced for further evaluation. The lead peptide **1** (VGDLTYLKK(FB), IC_50_ = 0.45 ± 0.06 μM, 25% stable in serum at 1 h) was further modified at the N-, C-, and bi-termini. C-terminal PEGylation increased the metabolic stability (>95% stable), but decreased binding affinity (IC_50_ = 3.7 ± 1 μM) was noted. C-terminal extension (**1i**, VGDLTYLKK(FB)KVART) significantly increased binding affinity for integrin α_v_β_6_ (IC_50_ = 0.021 ± 0.002 μM), binding selectivity for α_v_β_6_-expressing cells (3.1 ± 0.8:1), and the serum stability (>99% stable). Our results demonstrate the challenges in optimizing OBOC-derived peptides, indicate both termini of **1** are sensitive to modifications, and show that further modification of **1** is necessary to demonstrate utility as an ^18^F-peptide imaging agent.

## 1. Introduction

The expanding use of positron emission tomography (PET) imaging in oncology to aid in early detection, more accurate diagnosis, and patient treatment stratification has led to an increased demand of novel peptide-based molecular imaging agents [1]. Peptides are attractive as imaging agents for several reasons, including favorable pharmacokinetic properties, such as high target affinity, good tumor penetration, rapid non-specific clearance, and minimal immunogenicity [2]. Additionally, once peptide sequences are identified, they can be readily synthesized to high purity and are amenable to chemical modifications to alter affinity, charge, hydrophobicity, solubility, and stability [3]. The biological half-life of peptides ideally matches with many clinically used radionuclides, such as the short-lived positron emitting fluorine-18 (t_1/2_ = 109.7 min) that possesses favorable nuclear properties (e.g., 97% positron emission), and the ease of production with high yield and molar activity for PET imaging [4].

Combinatorial libraries, such as the one-bead one-compound (OBOC) and phage display, have provided indispensable tools for rapid identification of peptides for a given biological target, especially those expressed on the cell surface [5]. OBOC libraries display randomized peptide sequences on solid-support beads, which can be screened against either purified proteins or whole cells to identify receptor-specific peptides [6]. We, and others, have utilized the OBOC approach to identify peptides for the development of PET imaging agents [6,7]. However, to date, only a handful of OBOC-derived peptides, notably the α_5_β_1_–targeting OA02 peptide [8] and the α_4_β_1_-targeting LLP2A peptidomimetic [9,10], have been evaluated for preclinical in vivo imaging. Clearly, the current translation of OBOC-derived peptides from bench to bedside remains a slow process [11].

To ensure target-specificity of the identified peptides, on-bead library screening requires multiple rounds of screening to eliminate false positive (non-specific binders) [12]. This stringent and iterative screening of OBOC libraries for target-selective peptides is a lengthy, time-consuming, and inefficient process. For OBOC-derived peptides to be utilized as PET imaging agents, a positron-emitting isotope, such as fluorine-18, needs to be attached to the peptide. The introduction of the radioisotope, typically via a prosthetic group post-identification from the OBOC library, can have a negative impact on the pharmacokinetic properties and binding affinity of the original peptide, as it adds mass, alters hydrophobicity, and changes the charge of the peptide [13]. In addition, once a peptide sequence has been successfully identified, it typically requires further optimization before it can be used as an imaging agent, as peptides are susceptible to proteolytic degradation in serum in vivo, and are often eliminated rapidly from the system [14]. Therefore, chemical modifications of the OBOC-derived peptides are carried out to improve their in vivo stability to make them suitable for PET imaging.

In this work, we proposed to address some of the aforementioned limitations and describe the developmental process for the identification and optimization of integrin α_v_β_6_-targeting peptide PET imaging agents, using OBOC and on-bead two-color (OBTC) technologies. The integrin α_v_β_6_ is an epithelial-specific cell surface receptor that is undetectable in healthy adult epithelium, but is upregulated in many aggressive cancers, such as ovarian, colon, pancreatic, and breast, and its expression level has been linked to poor prognosis, making it an interesting target for both diagnostic and therapeutic agents [15]. Notable examples of integrin α_v_β_6_-targeting peptides that have been used as imaging agents include the A20FMDV2 [16] and Cystine Knot R_0_1 peptides [17]. Both peptides contain the known α_v_β_6_-targeting motif (DLXXL), originally identified from phage display [18], and they have been radiolabeled with ^18^F and ^64^Cu for in vivo imaging in small animal models [7,16,17,19,20]. In a proof of concept effort, Gagnon et al. reported the use of combinatorial libraries, solid-phase radiolabeling, and high throughput in vivo screening to evaluate an OBOC library featuring the DLXXL motif, and discovered 42 lead peptides in 11 consecutive days [7]. However, the identified lead peptides still required substantial optimization. 

Herein, we designed an OBOC library containing the known integrin α_v_β_6_-binding motif (DLXXL) [18] and the nonradioactive substitute of the commonly used ^18^F-radiolabeling prosthetic group, 4-fluorobenzoic acid ([^19^F]FBA) at the C-terminal lysine (XXDLXXLXK([^19^F]FB, 9-mer). To facilitate the direct identification of integrin α_v_β_6_-binding peptides in a single step, an OBTC cell screen assay [21], utilizing transfected fluorescent DX3puroβ_6_mCherry (α_v_β_6_+, red) and DX3puroEmGFP (α_v_β_6_−, green) cell lines, was investigated. Competitive binding ELISAs with purified integrins (α_v_β_1_, α_v_β_3_, α_v_β_5_, α_v_β_6_, and α_v_β_8_) were performed to evaluate the binding profiles of lead peptides. Furthermore, efforts focused on optimizing the most promising peptide via N-, C-, and bi-terminal chemical modifications such as methylation, acetylation, 4-fluorobenzoylation, and PEGylation, as well as C-terminal extension for improved binding affinity, binding selectivity, and metabolic stability. Peptides were radiolabeled on solid-phase using 4-[^18^F]fluorobenzoic acid ([^18^F]FBA) for evaluation of cell binding and serum stability.

## 2. Results

### 2.1. Design and Synthesis of 4-Fluorobenzoyl OBOC Library

**Scheme 1:**
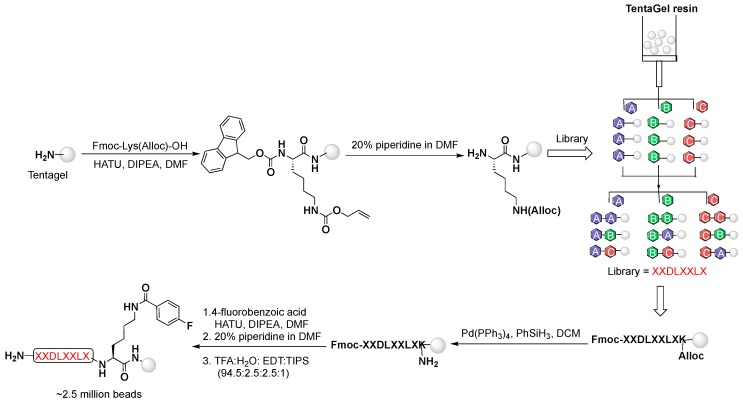
Design and synthesis of integrin α_v_β_6_-targeting 4-fluorobenzoyl-peptide library.

Using standard solid-phase peptide synthesis (SPPS) and the split-mix approach, the 9-mer peptide library of sequence “XXDLXXLXK([^19^F]FB)” was built containing the integrin α_v_β_6_-targeting motif (DLXXL) and the nonradioactive [^19^F]FBA, coupled to the sidechain of the built-in C-terminal lysine (K) at gram scale to yield approxmately 2.5 million unique sequences (theoretical permutation of 2.47 × 10^6^ peptides) (Scheme 1). 

### 2.2. On-Bead Two-Color (OBTC) Fluorescent Cell Screening Assay

**Figure 1:**
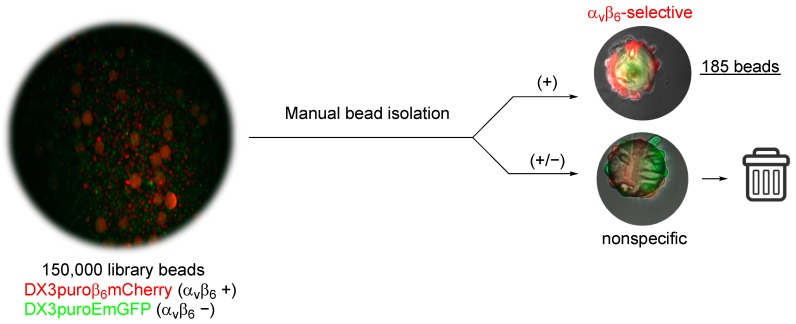
Schematic representation of the On-Bead Two-Color (OBTC) cell screening utilizing fluorescent DX3puroβ_6_mCherry (α_v_β_6_+, red) and DX3puroEmGFP (α_v_β_6_−, green) cells.

Dx3puroβ_6_mCherry (α_v_β_6_+, red) and DX3puroEmGFP (α_v_β_6_−, green) cells (500,000 total) were premixed in a petri dish for an hour prior to incubating with library beads (150,000) (Figure 1). A total of 185 beads were manually identified as integrin α_v_β_6_-specific (i.e., beads that were coated exclusively with the red cells (α_v_β_6_+)) and were isolated from the assay over 5 days. The first 5 beads isolated from the assay (**1**–**5**) were sequenced by Edman degradation and their sequences are shown in Table 1. 

### 2.3. On-Bead Validation of the Identified Peptide Sequences

**Figure 2:**
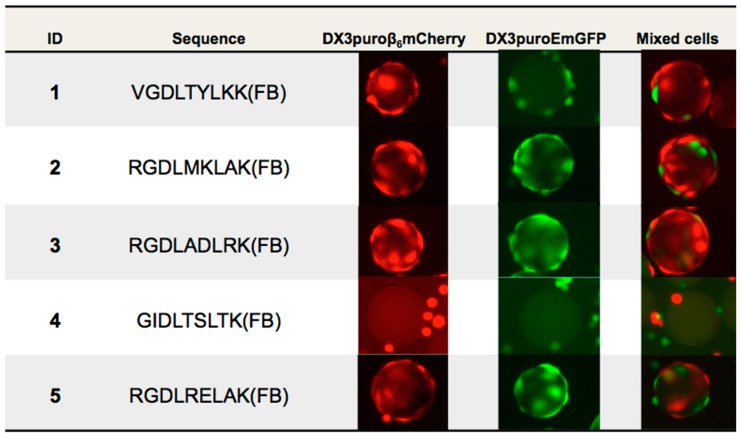
Representative pictures of **1**–**5** incubated with (**a**) DX3puroβ_6_mCherry (α_v_β_6_+, red); (**b**) DX3puroEmGFP (α_v_β_6_−, green); and (**c**) mixed DX3puroβ_6_mCherry (α_v_β_6_+, red), and DX3puroEmGFP (α_v_β_6_−, green) cells 3 h post-incubation.

In the mixed cell assay, all peptides showed good binding to the DX3puroβ_6_mCherry (α_v_β_6_+, red) cells and minimal cross binding to DX3puroEmGFP (α_v_β_6_−, green) cells, except **4**. In the single cell assay, **1** showed high selectivity for α_v_β_6_+, red cells with little binding to α_v_β_6_−, green cells, whereas **4** showed no binding to either cell line (Figure 2). **2**, **3**, and **5** also showed noticeable binding to DX3puroEmGFP (α_v_β_6_−, green) cells in the single cell assay. 

### 2.4. Competitive Binding ELISAs

As summarized in Table 1, all lead peptides, except for **4**, showed low micromolar IC_50_ values for α_v_β_6_, i.e., moderate to good binding to purified integrin α_v_β_6_. In summary, **1** was the most selective for integrin α_v_β_6_ (IC_50_ = 0.45 ± 0.06 μM), demonstrating a selectivity ratio of >200:1 when compared to the other integrins (α_v_β_1_, α_v_β_3_, and α_v_β_5_), and with some binding to integrin α_v_β_8_ (IC_50_ = 5.3 ± 0.14 μM, 20:1). The peptides containing the RGD motif (**2**, **3**, and **5**) all showed comparable affinity for integrin α_v_β_6_ (IC_50_ = 0.95 ± 0.17, 0.42 ± 0.07, and 0.89 ± 0.14 μM, respectively), however, with poor selectivity towards other integrins (α_v_β_1_, α_v_β_3_, and α_v_β_8_, except for α_v_β_5_ (>100:1)). Meanwhile, **4** consistently showed little to no binding to integrins α_v_β_6_ and α_v_β_3_, only achieving an IC_50_ > 100 μM.

### 2.5. Modifications of ***1***

Peptide **1** was selected for further optimization (Table 2). First, a series of N-terminal modifications were explored, including moving the 4-fluorobenzoyl moiety from the C-terminal lysine to the *N*-terminus (**1a**, IC_50_ = 0.81 ± 0.09 μM), N-terminal acetylation (**1b**, IC_50_ > 100 μM), N-terminal methylation (**1c**, IC_50_ > 5 μM), N-terminal PEGylation (**1d**, IC_50_ > 100 μM), and containing both the C- and N-terminal 4-fluorobenzoylation (**1e**, IC_50_ = 0.84 ± 0.36 μM). Modification of the *C*-terminus was also done via PEGylation (**1f**, IC_50_ = 3.7 ± 1 μM). In addition, the C-terminal PEGylated peptides were also synthesized as the *N*-acetylated (**1g**, IC_50_ > 100 μM) and *N*-fluorobenzoylated (**1h**, IC_50_ = 1.47 ± 0.23 μM) analogs. The *C*-terminus of **1** was also further extended by five amino acids, KVART, (**1i**, IC_50_ = 0.021 ± 0.002 μM). 

### 2.6. Radiochemical Synthesis of ^18^F-Radiolabeled Peptides

All radiolabeled peptides were obtained with molar activity of >37 GBq/μmol and radiochemical purity of >99% after semi-prep RP-HPLC purification, except for **2** (Table 3). Peptide **2** exhibited decomposition and formation of a radioactive by-product (22%) preceding the product peak (78%), as observed by analytical radio-RP-HPLC, even after semi-prep RP-HPLC purification (Figure S3). This by-product increased over time and as such **2** was not further evaluated.

### 2.7. In Vitro Evaluation of ^18^F-Radiolabeled Peptides

#### 2.7.1. In Vitro Cell Binding of ^18^F-Radiolabeled Peptides

**Figure 3:**
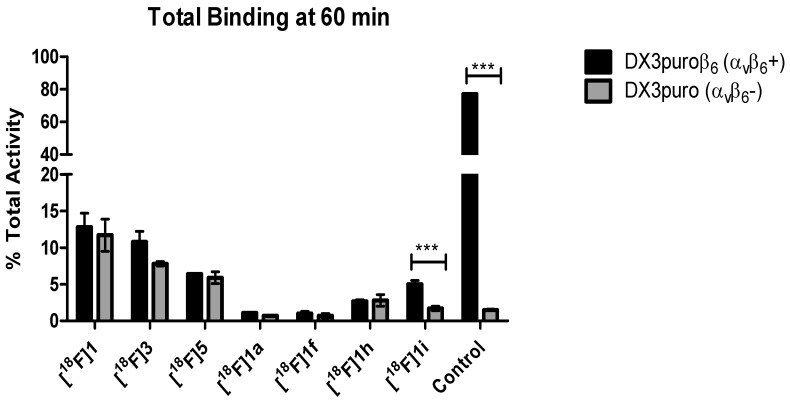
In vitro binding results of **[^18^F]1**, **[^18^F]3**, **[^18^F]5**, **[^18^F]1a**, **[^18^F]1f**, **[^18^F]1h**, **[^18^F]1i** and the control peptide after 60 min incubation with either DX3puroβ_6_ (α_v_β_6_+) or DX3puro (α_v_β_6_−) cell line (*n* = 3). *** paired Student *t*-test gives *p* < 0.05.

The control peptide [^18^F]FB-PEG_28_-A20FMDV2-K16R-PEG_28_ showed a typical binding level to DX3puroβ_6_ (α_v_β_6_+) cells (77.1 ± 0.1%) with minimal binding to DX3puro (α_v_β_6_−) cells (1.5 ± 0.1%), confirming the integrity of the binding assay (Figure 3) [22]. The % binding of **[^18^F]1**, **[^18^F]3**, and **[^18^F]5** to DX3puroβ_6_ (α_v_β_6_+) cells was 12.8 ± 1.9%, 7.7 ± 3.8%, 6.4 ± 0.1%, respectively. The % binding of **[^18^F]1**, **[^18^F]3**, and **[^18^F]5** to DX3puro (α_v_β_6_−) cells was 11.7 ± 2.2%, 5.3 ± 2.8%, and 5.9 ± 0.8%, respectively. Unfortunately, all three peptides exhibited non-favorable selectivity towards the DX3puroβ_6_ (α_v_β_6_+) cells, as demonstrated by low DX3puroβ_6_/DX3puro binding ratios (**[^18^F]1** = 1.1 ± 0.1:1, **[^18^F]3** = 1.4 ± 0.2:1, and **[^18^F]5** = 1.1 ± 0.1:1). Further, **[^18^F]1a**, **[^18^F]1f**, and **[^18^F]1h** all showed less than 2% binding to both DX3puroβ_6_ (α_v_β_6_+) and DX3puro (α_v_β_6_−) cell lines, and **[^18^F]1i** demonstrated 5.0 ± 0.5% binding to DX3puroβ_6_ (α_v_β_6_+) cells with a significant improvement in binding selectivity ratio of 3.1 ± 0.8:1 (*p* < 0.05). 

#### 2.7.2. Serum Stability of ^18^F-Radiolabeled Peptides

As summarized in Table 4, **[^18^F]1** remained 25% intact, but both **[^18^F]3** and **[^18^F]5** exhibited 100% degradation at 1 h post-incubation in mouse serum.

#### 2.7.3. Serum Stability of ^18^F-Radiolabeled Modified Derivatives of **1**

**Figure 4:**
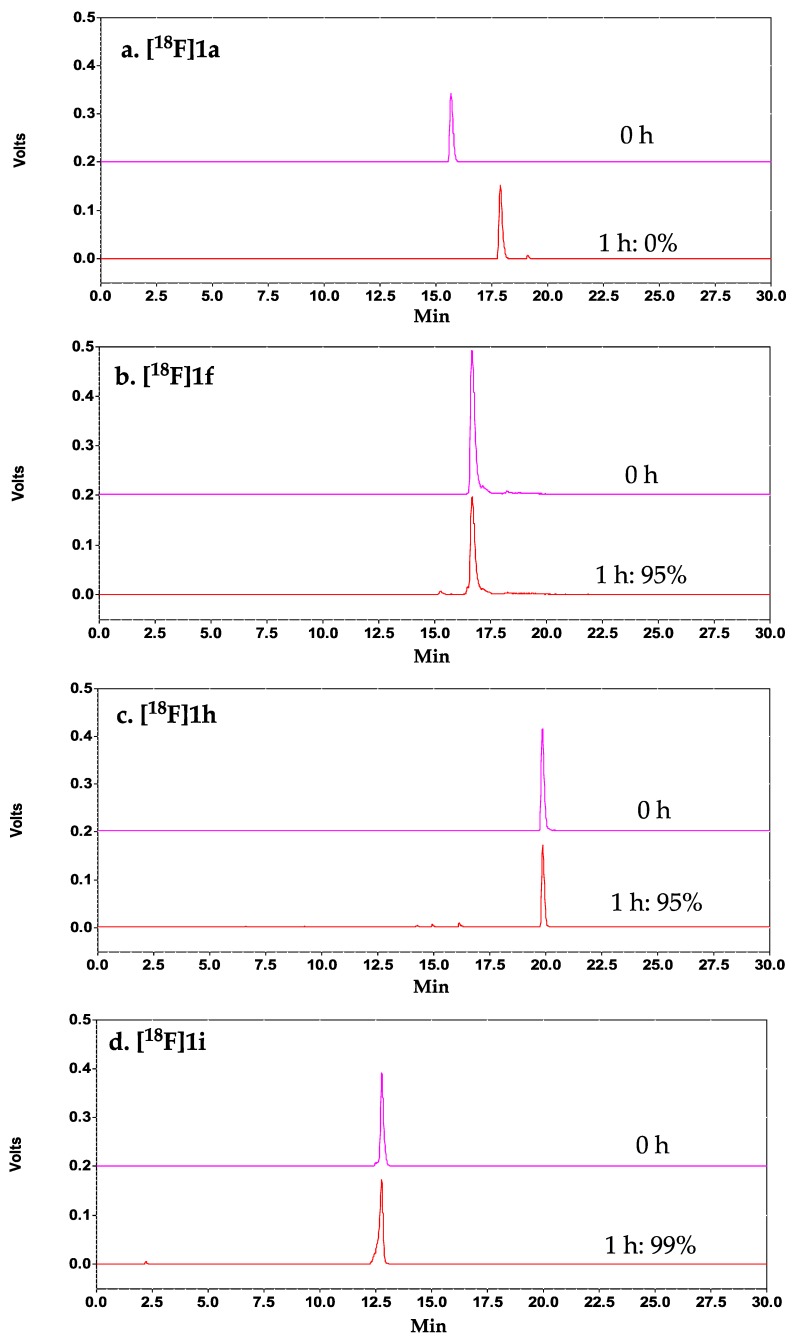
Radio-RP-HPLC chromatograms of the parent peptides (**a**) **[^18^F]1a**; (**b**) **[^18^F]1f**; (**c**) **[^18^F]1h**; and (**d**) **[^18^F]1i** (magenta) and their corresponding metabolites after 60 min incubating with mouse serum (red).

As indicated by radio-RP-HPLC, upon incubating with mouse serum for 1 h **[^18^F]1a** was completely metabolized; however, both **[^18^F]1f** and **[^18^F]1h** were >95% intact (Figure 4a–c), and **[^18^F]1i** remained >99% intact (Figure 4d). 

### 2.8. Evaluation of Secondary Structure of ***1***, ***1i***, and the Control Peptide Using Circular Dichroism (CD) Spectroscopy

**Figure 5:**
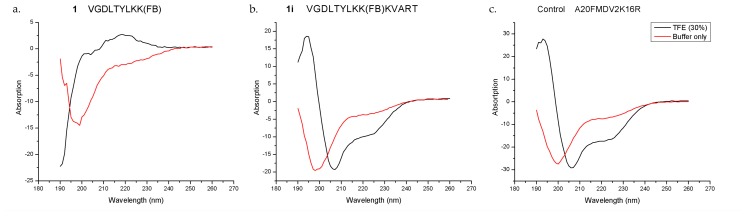
Circular dichroism (CD) spectra of (**a**) the parent peptide **1**; (**b**) **1i**; and (**c**) the control peptide in aqueous buffer (red) and in 30% TFE (black).

CD data revealed that all three peptides exhibited random coil conformation in CD buffer (red). However, α-helices were observed when 30% 2,2,2-trifluoroethanol (TFE) was added to CD buffer for **1i** (Figure 5b, black curve) and the control peptide (Figure 5c, black curve), whereas the parent peptide **1** did not form the helix under the same condition (Figure 5a).

## 3. Discussion

The OBOC technology has been successfully utilized to identify cancer-targeting peptides [6,7,8], yet the approach remains painstakingly slow, with clear delays between on-bead ligand identification and subsequent optimization to a potential imaging agent [7,11]. The aim of this work was to combine multiple strategies to streamline the development of integrin α_v_β_6_-targeting peptide-based imaging agents for PET. This included (1) OBOC chemistry to produce large peptide libraries, (2) elimination of setbacks due to post-screening modifications of peptides by incorporating 4-[^19^F]fluorobenzoic acid (FBA) into the peptide library design, (3) utilizing the OBTC fluorescent cell screening assay to expedite the on-bead library screening process (combine three rounds of screening into a single step), and (4) structural-based modifications to optimize OBOC-derived peptides for improved serum stability and pharmacokinetics properties. 

The incorporation of a prosthetic group or a chelator into a peptide sequence post-identification from a library can often have a negative impact on the binding properties [13]. To avoid this, the OBOC library was designed to contain the nonradioactive prosthetic group [^19^F]FBA pre-attached at the C-terminal lysine’s side chain. The placement of the prosthetic group at the *C*-terminus was designed to allow for Edman sequencing of the beads identified as selective binders to the cells that express integrin α_v_β_6_. In addition, the library contained the α_v_β_6_-targeting motif DLXXL, a motif originally discovered from a phage display library as an important binding sequence for the integrin α_v_β_6_ [18,23]. Stringent and iterative screening of OBOC libraries for target-selective peptides is lengthy, time-consuming, and not highly efficient. Traditionally, the OBOC libraries are screened against either purified proteins or whole cells, and positive beads (i.e., bound to protein or cells) are visualized under a microscope, and manually isolated [24]. To ensure target-specificity of the peptides, this process is often carried out in multiple rounds [24], with multiple washing cycles between subsequent rounds of screening, which often leads to broken or damaged beads. To streamline the screening, the OBTC cell-screening assay developed by Udagamasooriya et al. was adopted [21]. Using a mixed pool of two-color fluorescent cells differing only in the expression of integrin α_v_β_6_, beads that were recognized by the DX3puroβ_6_mCherry (α_v_β_6_+, red) cells were directly retrieved from the assay in one round of screening. This approach resulted in the rapid identification of 185 α_v_β_6_-positive beads from the entire 4-fluorobenzoyl peptide library (~2.5 million compounds), which otherwise would be difficult to achieve using the traditional screening approach. Conventionally, sequence determination of on-resin peptide is commonly carried out with Edman degradation [25,26,27]; however, this method is rather time consuming (hours to a day to sequence one peptide) and expensive (~$300/peptide) [28]. With these limitations in mind for the purpose of this work only, the first five beads identified from the first aliquot of screening were sequenced by Edman degradation for further evaluations. 

Of the five beads sequenced, three contained the three amino acids RGD sequence, a motif known to mediate cell adhesion and binds to α_v_ integrins, including integrin α_v_β_6_ [29]. The remaining two sequences did not share any homology and had the unique VGD and GID motifs. Qualitative on-bead cell binding with DX3puroβ_6_mCherry (α_v_β_6_+, red) cells confirmed binding of four of the five peptides. The three RGD containing peptides also showed some interactions with the negative cell line, which was likely due to recognition of the RGD motif by other RGD-binding integrins also expressed on the DX3puroEmGFP (α_v_β_6_−, green) cell line. This was corroborated by the ELISA data, where binding to all α_v_-integrins except for α_v_β_5_ was observed. The GID containing peptide did not bind to either cell line or α_v_-integrins by ELISA, and was possibly an outlier picked by mistake during the manual bead picking process. 

Based on the initial in vitro data, **1** (VGDLTYLKK(FB)) was selected for further optimization. Modifications at the *N*-terminus with various chemical moieties, including aliphatic (e.g., acetyl, methyl, PEG_12_) and aromatic (e.g., FBA) groups, PEGylation (e.g., PEG_28_) at the *C*-terminus, as well as a combination of both, were performed. Unfortunately, none of the modifications resulted in a new analog with a better binding affinity or selectivity for the integrin α_v_β_6_, as indicated by the ELISA and cell binding data. Interestingly, the *N*-terminus of the peptide appeared to be more tolerant towards modifications with the aromatic FBA than the aliphatic methyl, acetyl, and PEG moieties, which all showed poor binding (IC_50_ > 5–100 μM). These results suggest that the presence of the FB at the *N*-terminus of the peptide could possibly have favorable interactions with the protein residues in an existing binding pocket. A similar observation has been recently reported for a series of ^18^F-radiolabeled phosphoamidate peptidomimetic inhibitors for prostate specific membrane antigen (PSMA), where the FB ring of one of the analogues in the series engaged in an “arene-binding” interaction with the surface of the protein, and thus contributing to its high affinity in vitro and in vivo [30]. Future molecular docking studies or co-crystallization of these peptides with integrin α_v_β_6_ are necessary to provide better understanding of the peptide-integrin interaction.

Although peptides possess many favorable pharmacokinetics properties, such as high target affinity, good tumor penetration, and rapid non-specific clearance, they are prone to proteolytic degradation in vivo. PEGylation has been a widely adopted strategy to improve the pharmacokinetics properties of both small molecules and biopharmaceuticals (e.g., peptides, proteins, and antibodies, etc.) [31]. Based on our previous experience, the addition of PEG moieties to the *N*- and *C*-termini of the highly α_v_β_6_-selective A20FMDV2 peptide successfully prolonged the biological half-life, improved metabolic stability and enhanced tumor retention of the [^18^F]FB-PEG_28_-A20FMDV2, [^18^F]FB-(PEG_28_)_2_-A20FMDV2, and [^18^F]FB-PEG_28_-A20FMDV2-PEG_28_ in human tumor xenograft models [16,19,20]. Of the numerous peptides evaluated, **1** demonstrated the highest serum stability. To further improve the stability, C-terminal PEGylation of **1** was explored. Our results demonstrated that C-terminal PEGylation of the peptide (with and without N-terminal FBA, **1h**, and **1f**, respectively) indeed did significantly improve the metabolic stability of the peptide, with >95% intact peptide observed at 1 h post-incubation in mouse serum. Despite having excellent serum stability, both analogs showed significant reduction in binding affinity (<2% binding to DX3puroβ_6_ (α_v_β_6_+) cells). Our data suggests that the introduction of a PEG moiety at the *C*-terminus of the peptide could effectively shield the peptide from proteases, which is consistent with the trend observed for our [^18^F]FB-A20FMDV2-PEG_28_ (76% intact at 1 h) [19], comparing to the first generation [^18^F]FB-A20FMDV2 (0% intact) [16]. However, the introduction of the relatively large PEG moiety (MW = 1.5 kDa) to the relatively short peptide (MW = 1.1 kDa) resulted in a drastic loss in binding of **1f** and **1h** to integrin α_v_β_6_. A possible explanation is that the relatively large PEG chain length could have imposed some steric shielding effect on the peptide (i.e., PEG wrap around and shield the binding domain from interacting with the receptor), and in turn negatively affected the binding affinity of the resulting PEGylated peptides. This steric shielding effect of PEG on drug conjugates was previously reported by Mu et al. in a study investigating the impact of PEG chain length and PEGylation site on the bioactivity of the therapeutic protein Staphylokinase, using both experimental assays and computational simulations [32]. Future work entails the investigation of C-terminal PEGylation of **1** with shorter PEG chain length to further test this hypothesis. 

Since **1** (VGDLTYLKK(FB)) is a relatively small peptide, it is possible that modifications at either terminus of the peptide could negatively affect the binding affinity due to the close proximity to the binding domain. Furthermore, a co-crystal structure of the integrin α_v_β_6_ with pro-TGFβ3 peptide revealed that integrin α_v_β_6_ not only recognizes the RGD motif but also binds preferentially to the LXXL/I motif that folds into an amphipathic α-helix, fitting into a hydrophobic pocket composed solely from residues of the β_6_ subunit [33]. As reported previously, the α_v_β_6_-binding A20FMDV2 peptide has been confirmed to have a post-RGD α-helix, contributing to its high affinity and selectivity for integrin α_v_β_6_ [34]. Additionally, other α_v_β_6_-selective peptides reported in the literature, such as the R_0_1 peptide [17] and the H2009.1 peptide [23], are relatively larger, composing of 30- and 20-amino acids, respectively. To that end, we suspected that the lack of binding affinity and selectivity of our peptide **1** (9-mer) to integrin α_v_β_6_ could be attributed to its inability to form a stabilized α-helix to fit the binding pocket tightly. This led us to explore extending the *C*-terminus of **1** to improve its binding affinity and selectivity for integrin α_v_β_6_. Indeed, extension of the *C*-terminus of **1** with the KVART sequence from the known integrin α_v_β_6_-binding A20FMDV2 (NAVPNLRGDLQVLAQKVART) resulted in a 14-mer peptide with improved binding affinity and a 3-fold increase in binding selectivity for DX3puroβ_6_ (α_v_β_6_+) cells, and excellent serum stability (>99% intact at 1 h). The CD results demonstrated that both the control A20FDMV2 and the 14-mer **1i** peptides could form an α-helix in 30% TFE/buffer, while the parent peptide **1** could not under the same condition, which supported our hypothesis on the importance of peptide secondary structure on binding affinity and selectivity for integrin α_v_β_6_.

## 4. Materials and Methods

General Experimental Information. All the Fmoc-protected amino acids were purchased from GL Biochem (Shanghai, China). TentaGel S NH_2_ resin (90 μm) was purchased from RAPP Polymere GmbH (Tuebingen, Germany) and NovaSyn TGR resin was purchased from NovaBiochem (Burlington, MA, USA). All other reagents were purchased from Sigma Aldrich (St. Louis, MO, USA) and Fluka (Mexico City, Mexico), unless otherwise specified. A reverse-phase high performance liquid chromatography (RP-HPLC) Beckman Coulter Gold system (Brea, CA, USA) was used for peptide characterization (Phenomenex Jupiter 4 μ Proteo 90 Å column, Torrance, CA, USA) and purification (Phenomenex Jupiter 10 μ C18 300 Å column). The mobile phase used was 0.05% trifluoroacetic acid (TFA) in water (*v*/*v*; solvent A) and 100% acetonitrile (solvent B); solvent B isocratic 9% for 2 min followed by a linear gradient to 81% over 30 min at a flow rate of 1.5 mL/min (analytical RP-HPLC) and 3 mL/min (semi-prep RP-HPLC). All peptides were characterized using a UltraFlextreme (Bruker, Billerica, MA, USA) Matrix-assisted laser desorption-ionization time of flight/time of flight (MALDI TOF/TOF) mass spectrometer with a matrix of sinapinic acid or α-cyano-4-hydroxycinnamic acid.

### 4.1. OBOC Library Synthesis

The peptide library was assembled on TentaGel resin using the “split-mix” technique and standard Fmoc-based solid phase peptide synthesis (SPPS), utilizing all l-amino acids except l-cysteine. Briefly, the resin was first divided into 19 aliquots; each was coupled with a single amino acid (3 equiv.) using *O*-(7-azabenzotriazol-1-yl)-*N*,*N*,*N*′,*N*′-tetramethyluronium hexafluorophosphate (HATU, 2.7 equiv.) as a coupling agent and *N*,*N*-diisopropylethylamine (DIPEA, 3 equiv.) as a base in *N*,*N*-dimethylformamide (DMF) at room temperature for 1.5 h. At the end of each coupling, the resin was re-pooled, mixed, and split for the next coupling step. Fluorenylmethyloxycarbonyl (Fmoc)-protecting group was removed with 20% piperidine in DMF, and allyloxycarbonyl (Alloc)-protecting group was removed with tetrakis(triphenylphosphine)palladium(0) (Pd(PPh_3_)_4_, 0.5 equiv.), and phenylsilane (PhSiH_3_, 20 equiv.) in dichloromethane (DCM). Global deprotection was achieved with trifluoroacetic acid (TFA) mixture with triisopropylsilane (TIPS) and water (95:2.5:2.5 *v*/*v*/*v*) for 3 h. Finally, the resin was washed thoroughly with DMF, water, and methanol (10× each) and stored in 70% ethanol at 4 °C until screening. 

### 4.2. Peptide Synthesis

All peptides were synthesized manually on NovaSyn TGR resin (0.25 mmol/g, Burlington, MA, USA) using standard Fmoc-based SPPS yielding C-terminal amides. Starting with *N*-α-Fmoc-*N*-ε-1-(4,4-dimethyl-2,6-dioxocyclohex-1-ylidene)-3-methylbutyl-l-lysine (Fmoc-Lys(ivDde)-OH), orthogonal protecting group for site-specific labeling, each amino acid was coupled using HATU (2.7 equiv.) as a coupling agent and DIPEA (3 equiv.) as a base in DMF at room temperature for 1.5 h. The removal of the Fmoc- and ivDde-protecting groups was facilitated with 20% piperidine in DMF and 2% hydrazine in DMF, respectively. Peptides were cleaved off the resin using a mixture of TFA, water, and TIPS (95:2.5:2.5 *v*/*v*/*v*) for 3 h. The resin was washed with fresh TFA and the filtrate was collected then evaporated under reduced pressure. Crude peptides were precipitated in diethyl ether and extracted from the water/ether bilayer. All crude peptides were purified on the semi-prep HPLC. The purified peptides were analyzed on the analytical HPLC and characterized by MALDI-TOF/MS.

### 4.3. Cell Lines

DX3Puro (α_v_β_6_−) and DX3Puroβ_6_ (α_v_β_6_+) have been described previously [35,36] and were a generous gift from Dr. John Marshall. To generate DX3puroEmGFP and DX3puroβ_6_mCherry, DX3Puro and DX3Puroβ_6_ cells were transduced with lentivirus carrying EmGFP or mCherry (under control of an EF1α promoter) and the blasticidin resistance gene (under control of a hybrid SV40/EM7 promoter). Transduced cells were cultured at ultra-low density in Dulbecco’s Modified Eagle Medium (DMEM, Gibco, Waltham, MA, USA), supplemented with 10% FBS and blasticidin (10 µg/mL) at 37 °C in the presence of 5% CO_2_ for 21 days. Clonal populations of DX3puroEmGFP and DX3puroβ_6_mCherry were then isolated, expanded, and screened for fluorescence, and in the case of DX3puroβ_6_mCherry, retention of ITGβ_6_ expression. Integrin expression was determined by flow cytometry.

### 4.4. Flow Cytometry

Subconfluent cells were washed with PBS and harvested with Trypsin-EDTA (0.25%, Phenol-red, Gibco). Cells were treated once with PBS containing 0.1% BSA and 0.1% sodium azide (wash buffer), counted by trypan blue exclusion and resuspended at 6 × 10^6^ cells/mL. Aliquots of cells (50 μL) were treated with primary antibody clone 10D5 (Millipore Sigma, Burlington, MA, USA, at 10 µg/mL in serum free DMEM, 50 μL) for 60 min at 4 °C and washed twice with wash buffer (WB). AlexaFluor488 goat anti-mouse secondary antibody (ThermoFisher, Waltham, MA, USA, 1:50 dilution in serum free DMEM, 50 μL) was applied to the cells for 30 min at 4 °C. Cells were washed twice, resuspended in 0.5 mL WB, and scanned on the LSRFortesa Flow Cytometer (Becton Dickinson, Franklin Lakes, NJ, USA) and analyzed using FACSDiva software (8.02, San Jose, CA, USA), acquiring 1 × 10^4^ events.

### 4.5. Library Screening

#### 4.5.1. Library Beads Preparation

Prior to screening with cells, the beads were thoroughly washed to remove all traces of ethanol. An aliquot of beads (200 μL, equivalent to approximately 150,000 beads) was dried overnight, washed over vacuum with deionized water (10×), allowed to swell in water for 2 to 4 h at room temperature, followed by washing with PBS (5×), and suspended in PBS for 15 min.

#### 4.5.2. OBTC Fluorescent Cell Screening

DX3puroEmGFP (α_v_β_6_−) and DX3puroβ_6_mCherry (α_v_β_6_+) cells were cultured in DMEM supplemented with 10% FBS, 1% PSG, and blasticidin (10 µg/mL) as adherent monolayers at 37 °C in the presence of 5% CO_2_ in a humidified incubator for 3 days. At 80% confluency, cells were harvested, counted, and re-suspended in 10% fetal bovine serum (FBS) phenol red–free DMEM for screening. Briefly, cells (500,000 cells/cell line) were mixed in 20 mL phenol red-free DMEM in a non-treated tissue culture petri dish (100 mm; Spectrum Chemicals and Laboratory Products) for 1 h. Library beads (150,000) were added to the mixed cells and incubated at 37 °C with gentle mixing for 3 h. After incubation, the beads were analyzed under a fluorescent microscope. Beads that were >80% covered in DX3puroβ_6_mCherry cells (α_v_β_6_-specific) were manually selected with a micropipette. The α_v_β_6_-specific beads isolated from the assay were sequenced using Edman degradation.

### 4.6. Competitive Binding ELISAs

Competitive binding ELISAs were performed to evaluate the binding affinity of each peptide relative to each integrin’s biotinylated natural ligand (transforming growth factor beta 1 latency-associated peptide or TGFβ1-LAP (G&P Biosciences, Santa Clara, CA, USA) for integrins α_v_β_6_ and α_v_β_8_, fibronectin (Thermo Fisher, Waltham, MA, USA) for integrin α_v_β_1_, and vitronectin (Thermo Fisher) for integrins α_v_β_3_ and α_v_β_5_), as previously described [22]. In brief, anti-α_v_ antibody (P2W7, 5 μg/mL, Abcam) was plated (50 μL/well) at 37 °C for 1 h, washed with PBS (3×), and blocked overnight with blocking buffer (300 μL/well, 5% bovine serum albumin (BSA), 1% Tween 20, in PBS), followed by washing with PBS (3×). Purified integrin (R&D Systems, Minneapolis, MN, USA) in conjugate buffer (50 μL/well, 20mM Tris, 1mM MnCl_2_, 150 mM NaCl, 0.1% Tween, 1% BSA in water) was then added to each well, incubated at 37 °C for 1 h, followed by washing using wash buffer (20 mM Tris, 1 mM MnCl_2_, 150 mM NaCl, 0.1% Tween in water, 3×). Serial dilutions of each peptide (10 μM–10 pM or 100 μM–100 pM in conjugate buffer) and each integrin’s respective biotinylated natural ligand was added to each well (a total volume of 50 μL/well). The plate was incubated at 37 °C for 1 h, then washed with wash buffer (3×). A 1:1000 dilution of ExtrAvidin Horseradish Peroxidase (HRP) was added to each well (50 μL/well), incubated at 37 °C for 1 h, and then washed with wash buffer (3×). The ExtrAvidin HRP was detected with 3,3′,5,5′-tetramethylbenzidine (50 μL/well, Promega, Madison, WI, USA) for 10–15 min at room temperature. The reaction was stopped by adding 1N H_2_SO_4_ (50 μL/well) and the absorbance was measured in a Multiscan Ascent plate reader (Thermo Fisher) at 450 nm. Half-maximal inhibitory concentration (IC_50_) of peptides was determined fitting to sigmoidal dose-response model in GraphPad Prism 5.0 (San Diego, CA, USA).

### 4.7. Radiochemical Synthesis

All peptides were radiolabeled with [^18^F]fluorobenzoic acid ([^18^F]FBA) on resin following our well-established solid-phase radiolabeling protocols [16,20,37]. Briefly, [^18^F]fluoride was reacted with ethyl-4-(trimethylammonium triflate)benzoate in the presence of 4,7,13,16,21,24-hexaoxa-1,10-diazabicyclo [8.8.8]hexacosane (Kryptofix^TM^ [K2.2.2], (Sigma Aldrich, St. Louis, MO, USA) and potassium carbonate at 100 °C in dimethylsulfoxide (DMSO) for 15 min, followed by saponification of the ethyl ester protect group to yield [^18^F]FBA. The [^18^F]FBA was re-dissolved in DMF and coupled to the peptide (~5 mg resin) using HATU (5 mg)/DIPEA (10 μL). The ^18^F-radiolabeled peptide on resin was cleaved and globally deprotected with trifluoroacetic acid/triisopropylsilane/water 95:2.5:2.5 (*v*/*v*/*v*) mixture for 30 min at room temperature. The crude peptide was purified by semi-prep radio-RP-HPLC (C18-column) and formulated in PBS for in vitro assays. The purity and molar activity of the ^18^F-radiolabeled peptide was confirmed via analytical radio-RP-HPLC. Identity of the ^18^F-radiolabeled peptide was confirmed by co-injection of the ^19^F-peptide, and all analytical radio-RP-HPLC analysis was performed for each ^18^F-radiolabeled peptide prior to use.

### 4.8. The ^18^F-Radiolabeled Peptide Cell Binding Assay

Cell binding assay was performed as previously described [19,20]. In brief, each cell line was suspended at a concentration of 75 × 10^6^ cells/mL in serum-free medium and aliquots of each cell line (3.75 × 10^6^ cells in 50 μL) was added to 1.5 mL microcentrifuge tubes previously blocked with 5% BSA in PBS for 10 min at room temperature. The ^18^F-radiolabeled peptide was prepared in a stock solution of 148 kBq in 1 mL PBS (pH 7.2). Aliquots of each ^18^F-radiolabeled peptide (50 μL, 7.4 kBq per sample) were added to each cell sample and incubated at 37 °C for 1 h with frequent suspension. After incubation, each sample was centrifuged and the supernatant was collected. The cell pellet was washed with 500 μL PBS, centrifuged, and the supernatant was combined with the previously collected supernatant samples. The cell pellet was resuspended in 600 μL PBS and transferred to gamma counter tubes. All pellet and supernatant samples were measured in a Wizard 1470 gamma counter (Perkin Elmer, Waltham, MA, USA). Cell binding percentage for each cell line was calculated as the radioactivity associated, with pellet divided by the total radioactivity associated with both the pellet and supernatant.

### 4.9. Serum Stability Assay

Peptide stability was evaluated in mouse serum as previously described [19,20]. In brief, formulated ^18^F-radiolabeled peptide (~3.7 MBq in PBS) was incubated with mouse serum (500 μL, Sigma Aldrich, St. Louis, MO) at 37 °C for 1 h. An aliquot of ^18^F-radiolabeled peptide in mouse serum (100 μL) was taken at 1 h, mixed with absolute ethanol (500 μL, 4 °C), and centrifuged to precipitate serum protein. The supernatant was diluted with HPLC solvent A before radio-HPLC analysis to determine the fraction of intact ^18^F-radiolabeled peptide. 

### 4.10. Circular Dichroism (CD) Spectroscopy

CD spectroscopy was performed on a Jasco J-810 spectropolarimeter equipped with a Jasco CDF-426S Peltier set to 25 °C (Easton, MD, USA). Lyophilized peptide was diluted to 0.2 mg/mL in buffer (final concentration was 25 mM phosphate + 100 mM NaF in PBS, pH 7.4), with or without 30% TFE (*v*/*v*), placed in a quartz cuvette (1 mm), and after extensive purging with nitrogen, scanned in the region 190–260 nm (scan speed was 20 nm/min). An average of five scans were baseline-subtracted (buffer, 25 mM phosphate + 100 mM NaF in PBS). 

### 4.11. Data and Statistical Analysis

Statistical analysis and comparisons between groups were performed using the student *t* test. A *p* value of 0.05 or less was considered statistically significant. 

## 5. Conclusions

In conclusion, this work utilized the OBOC technology, applied the OBTC fluorescent cell screening assay, and provided a framework for optimization of OBOC-derived peptides to streamline the development of ^18^F-peptide based imaging agents for PET. To expedite the development of peptide identified from OBOC library into useful imaging agents, we eliminated the need of post-screening modification by designing a 4-fluorobenzoyl peptide library, improved the traditional on-bead manual library screening using OBTC fluorescent cell-screening, and further improved the binding affinity, selectivity, and serum stability of the lead peptide using numerous strategies, including N-, C-, and bi-terminal modifications. Chemical modifications for the optimization of an identified peptide sequence have many challenges, as the outcome of each modification will depend on the peptide sequence and the target receptor, and their behavior cannot be predicted. It is fair to conclude that optimizing multiple parameters (i.e., binding affinity, selectivity, and serum stability) of a peptide identified from a library to be used as an imaging agent remains a daunting task. 

## Figures and Tables

**Table 1:** ELISA data showing the relative binding affinity and selectivity of lead peptides measured by IC_50_ values (*n* = 3).

ID	Sequence	IC_50_ (μM)				
		α_v_β_1_	α_v_β_3_	α_v_β_5_	α_v_β_6_	α_v_β_8_
**1**	VGDLTYLKK(FB)	>100	>100	>100	0.45 ± 0.06	5.3 ± 0.14
**2**	RGDLMKLAK(FB)	0.9 ± 0.12	2.2 ± 0.83	>100	0.95 ± 0.17	2.6 ± 0.35
**3**	RGDLADLRK(FB)	0.6 ± 0.15	0.5 ± 0.08	>100	0.42 ± 0.07	0.2 ± 0.02
**4**	GIDLTSLTK(FB)	ND	>100	ND	>100	ND
**5**	RGDLRELAK(FB)	1.9 ± 0.3	0.3 ± 0.02	>100	0.89 ± 0.14	2.6 ± 0.29

ND = not determined.

**Table 2:** ELISA data showing the relative binding affinity of derivatives of **1** to integrin α_v_β_6_ measured by IC_50_ values (*n* = 3).

ID	Sequence	IC_50_ (μM) α_v_β_6_
1 *	VGDLTYLKK(FB)	0.45 ± 0.06
1a *	FB-VGDLTYLKK	0.81 ± 0.09
**1b**	Ac-VGDLTYLKK(FB)	>100
**1c**	Me-VGDLTYLKK(FB)	>5
**1d**	PEG_12_-VGDLTYLKK(FB)	>100
**1e**	FB-VGDLTYLKK(FB)	0.84 ± 0.36
1f *	VGDLTYLKK(FB)-PEG_28_	3.7 ± 1
**1g**	Ac-VGDLTYLKK(FB)-PEG_28_	>100
1h *	FB-VGDLTYLKK(FB)-PEG_28_	1.47 ± 0.23
1i *	VGDLTYLKK(FB)KVART	0.021 ± 0.002

Note: * peptides that were ^18^F-radiolabeled for cell binding and serum stability studies.

**Table 3:** Analytical data of all ^18^F-radiolabeled peptides evaluated in cell binding and serum stability experiments.

ID	Sequence	dc RCY (%)	RCP (%)	Retention Time (min)
**[^18^F]1**	VGDLTYLKK([^18^F]FB)	5.4 ± 1.2	>99	14.2
**[^18^F]2**	RGDLMKLAK([^18^F]FB)	4	78	13.6
**[^18^F]3**	RGDLADLRK([^18^F]FB)	13.4	>99	12.5
**[^18^F]5**	RGDLRELAK([^18^F]FB)	13.2 ± 10.1	>99	12.7
**[^18^F]1a**	[^18^F]FB-VGDLTYLKK	6.5	>99	15.7
**[^18^F]1f**	VGDLTYLKK([^18^F]FB)-PEG_28_	12.8	>95	16.7
**[^18^F]1h**	FB-VGDLTYLKK([^18^F]FB)-PEG_28_	10.9	>99	19.8
**[^18^F]1i**	VGDLTYLKK([^18^F]FB)KVART	2.8	>99	12.8

**Table 4:** Summary of the metabolic stability of ^18^F-radiolabeled lead peptides evaluated in mouse serum 1 h post-incubation.

ID	Sequence	% Intact (1 h)
**[^18^F]1**	VGDLTYLKK([^18^F]FB)	25
**[^18^F]3**	RGDLMKLAK([^18^F]FB)	0
**[^18^F]5**	RGDLADLRK([^18^F]FB)	0

## Supplementary Materials

The following are available online. Table S1: Analytical data of synthetic peptides showing retention time, purity, and mass confirmation; Table S2: Analytical Radio-RP-HPLC chromatogram confirming the purity of [^18^F]peptides; Figure S1: Analytical RP-HPLC chromatograms confirming the purity of synthetic peptides; Figure S2: Mass analysis of synthetic peptides using MALDI/MS; Figure S3: Radio-RP-HPLC of [^18^F]peptides showing the radioactive PMT trace (red) and their co-injected [^19^F]peptide standards (green UV220nm).
