# Supplementary material for: Identification, Characterization, and Optimization of Integrin αvβ6-Targeting Peptides from a One-Bead One-Compound (OBOC) Library: Towards the Development of Positron Emission Tomography (PET) Imaging Agents
Source: Molecules. 2019 Jan 16;24(2):309. doi: 10.3390/molecules24020309 (PMC6359284; doi:10.3390/molecules24020309)

molecules

MDPI

SUPPLEMENTARY MATERIALS FOR

Identification, Characterization, and Optimization of Integrin $\alpha_v\beta_6$-Targeting Peptides from a One-Bead One-Compound (OBOC) Library: Towards the Development of Positron Emission Tomography (PET) Imaging Agents

Yng (Sarah) C. Tang [1], Ryan A. Davis [2], Tanushree Ganguly [1] and Julie L. Sutcliffe [1,2,3,4,*]

[1] Department of Internal Medicine, Division of Hematology/Oncology, University of California, Davis and Sacramento, CA 95817, USA; yngtang@ucdavis.edu (Y.C.T.); tganguly@ucdavis.edu (T.G.)
[2] Department of Biomedical Engineering, University of California, Davis, CA 95616, USA; rydavis@ucdavis.edu
[3] Center for Molecular and Genomic Imaging, University of California, Davis, CA 95616, USA
[4] Radiochemistry Research and Training Facility, University of California, Davis, Sacramento, CA 95817, USA
* Correspondence: jlsutcliffe@ucdavis.edu; Tel.: +1-916-734-5536

CONTENTS

Supplementary Figures

Table S1. Analytical data of synthetic peptides showing retention time, purity and mass confirmation ……………………………………………………………………………….S2
Table S2. Analytical Radio-RP-HPLC chromatogram confirming the purity of [^{18}F]peptides……………………………………………………………………………….S8
Figure S1. Analytical RP-HPLC chromatograms confirming the purity of synthetic peptides………………………………………………………………………………….S3
Figure S2. Mass analysis of synthetic peptides using MALDI/MS …………………………....S4
Figure S3. Radio-RP-HPLC of [^{18}F]peptides showing the radioactive PMT trace (red) and their co-injected [^{19}F]peptide standards (green UV220nm)…………………………………………………..…………………..………S8

SUPPLEMENTARY TABLE S1. Analytical data of all synthetic peptides showing retention time, purity and mass confirmation.

ID	Sequence	t_R [min]	Purity [%]	Mass (g/mol) Calc.	Mass (g/mol) Obs.
**1**	VGDLTYLKK(FB)	14.2	>96	1156. 6	1157.6
**2**	RGDLMKLAK(FB)	13.0	>99	1151. 6	1152.6
**3**	RGDLADLRK(FB)	12.5	>99	1163.6	1164.5
**4**	GIDLTSLTK(FB)	15.7	>97	1067.5	1090.7
**5**	RGDLRELAK(FB)	12.6	>99	1177.6	1178.5
**1a**	FB-VGDLTYLKK	15.1	>99	1156.6	1157.6
**1b**	Ac-VGDLTYLKK(FB)	16.5	>99	1198.6	1199.8
**1c**	Me-VGDLTYLKK(FB)	14	>99	1170.6	1171.7
**1d**	PEG_{12}-VGDLTYLKK(FB)	15.6	>99	1756.0	1757.1
**1e**	FB-VGDLTYLKK(FB)	19	>99	1278.6	1279.6
**1f**	VGDLTYLKK(FB)-PEG_{28}	15.4	>99	2460.4	2461.4
**1g**	Ac-VGDLTYLKK(FB)-PEG_{28}	17.7	>95	2502.4	2503.8
**1h**	FB-VGDLTYLKK(FB)-PEG_{28}	19.7	>99	2582.4	2583.6
**1i**	VGDLTYLKK(FB)KVART	12.8	>99	1711.9	1713.0

SUPPLEMENTARY FIGURE S1

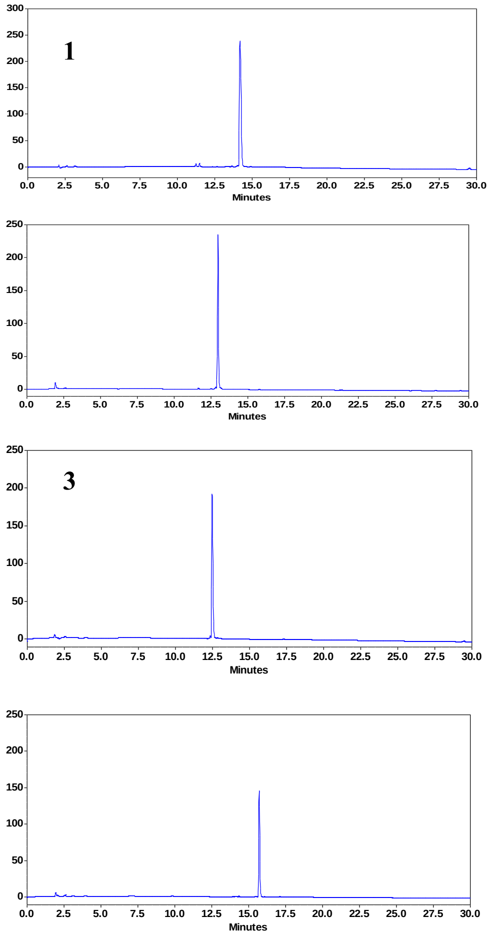

2

4

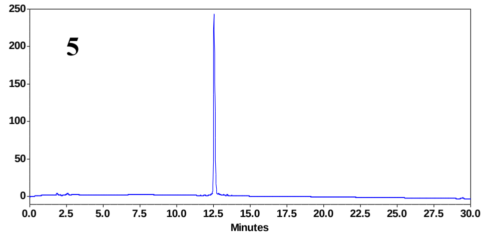

1a

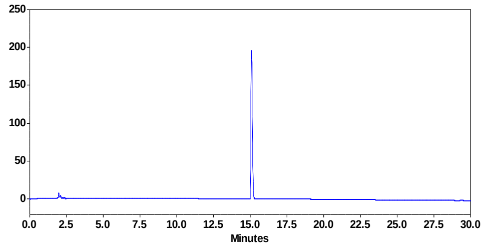

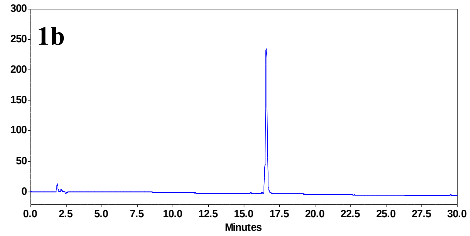

1c

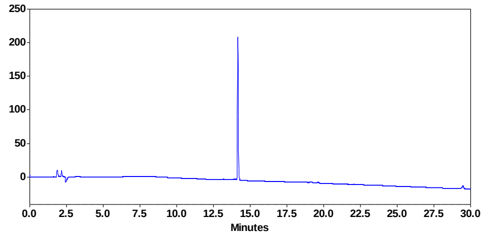

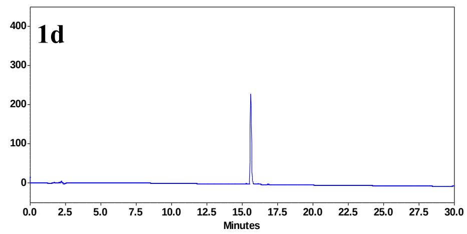

1e

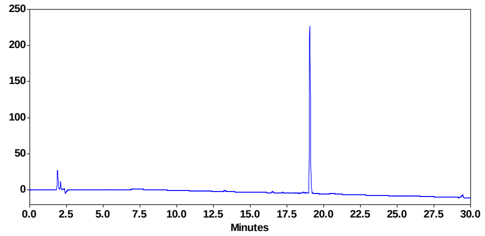

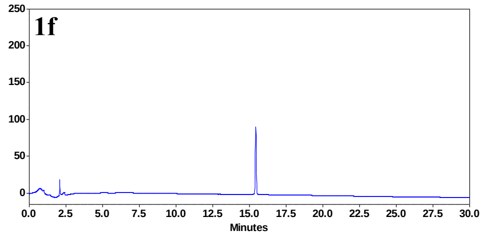

1g

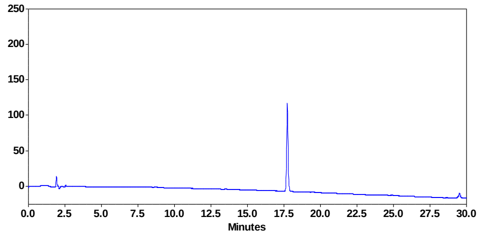

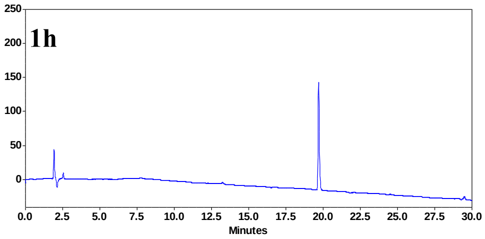

1i

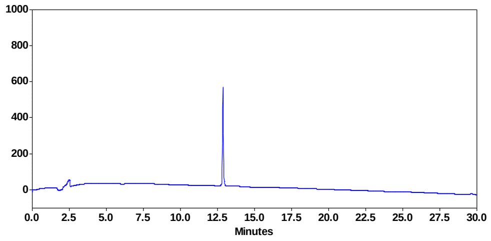

SUPPLEMENTARY FIGURE S2.

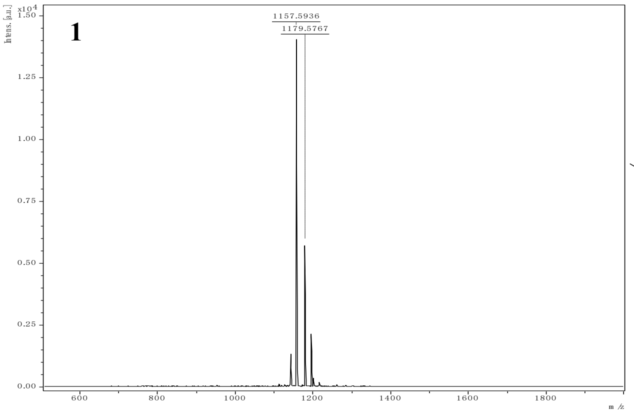

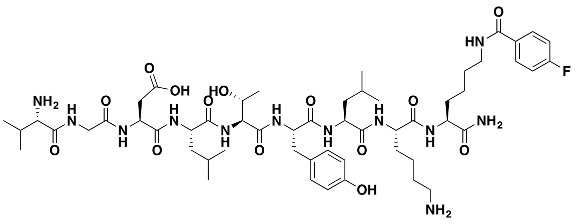

1

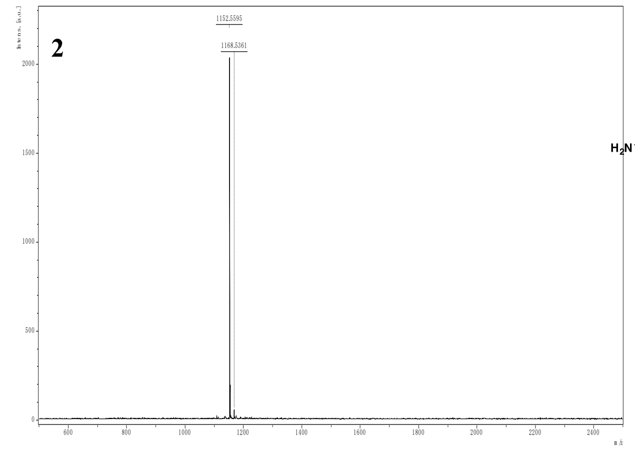

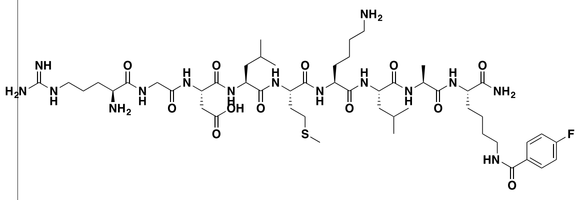

2

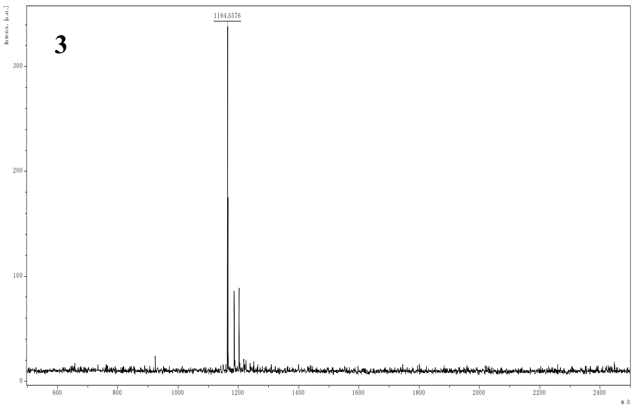

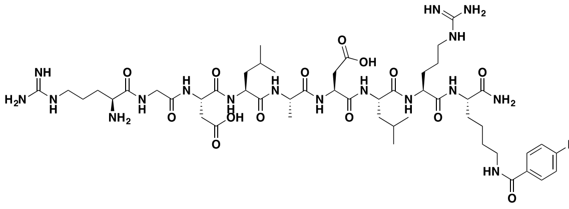

3

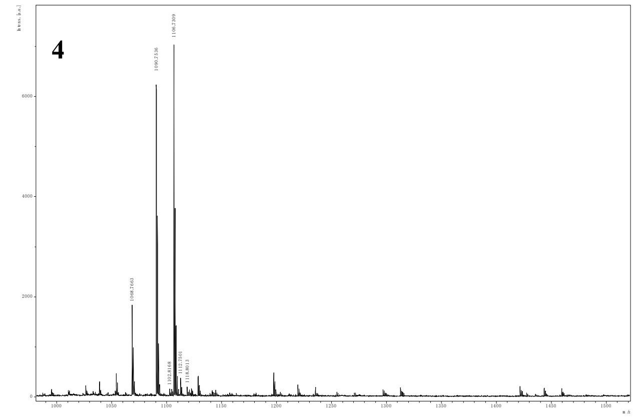

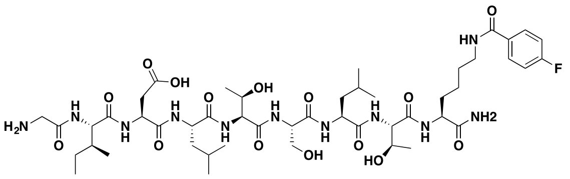

4

SUPPLEMENTARY FIGURE S2 (CONTINUED)

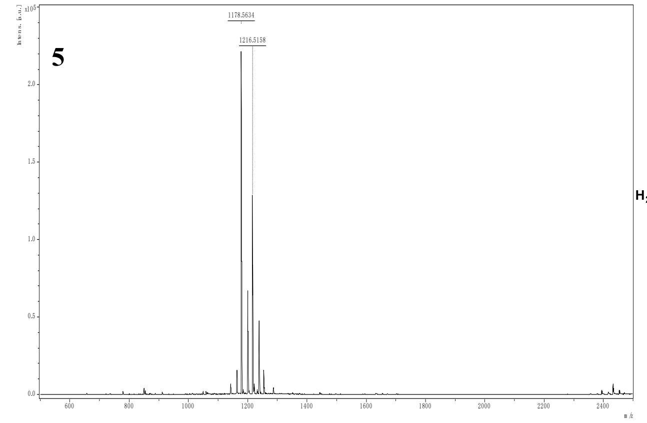

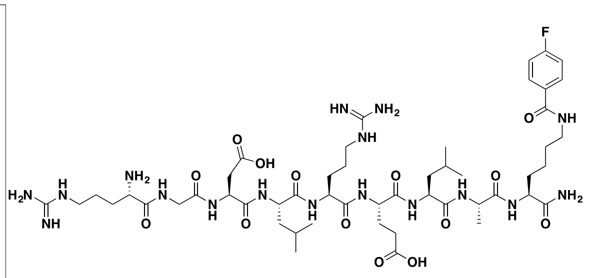

5

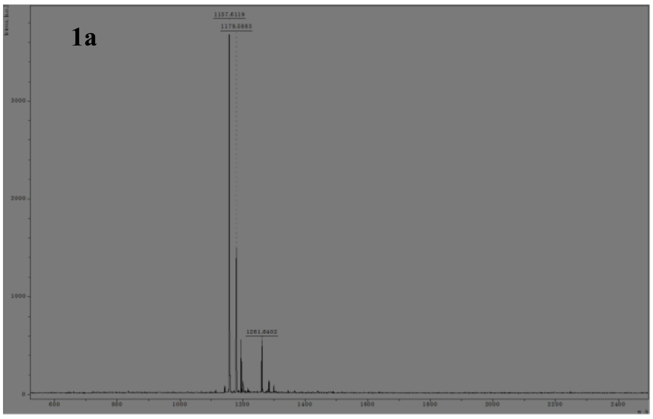

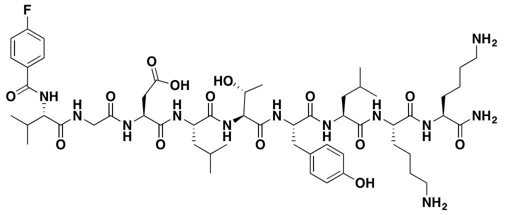

1a

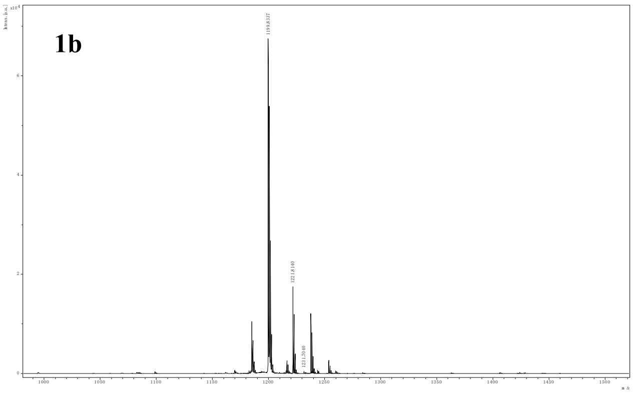

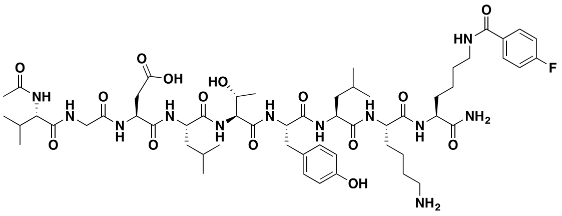

1b

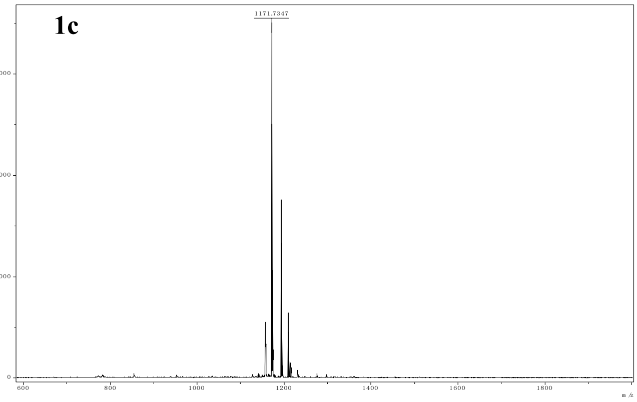

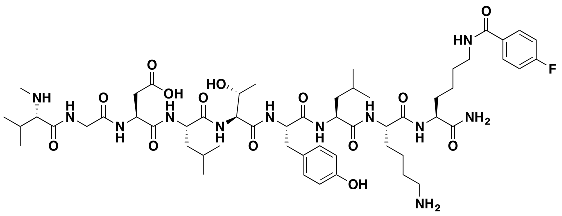

1c

SUPPLEMENTARY FIGURE S2 (CONTINUED)

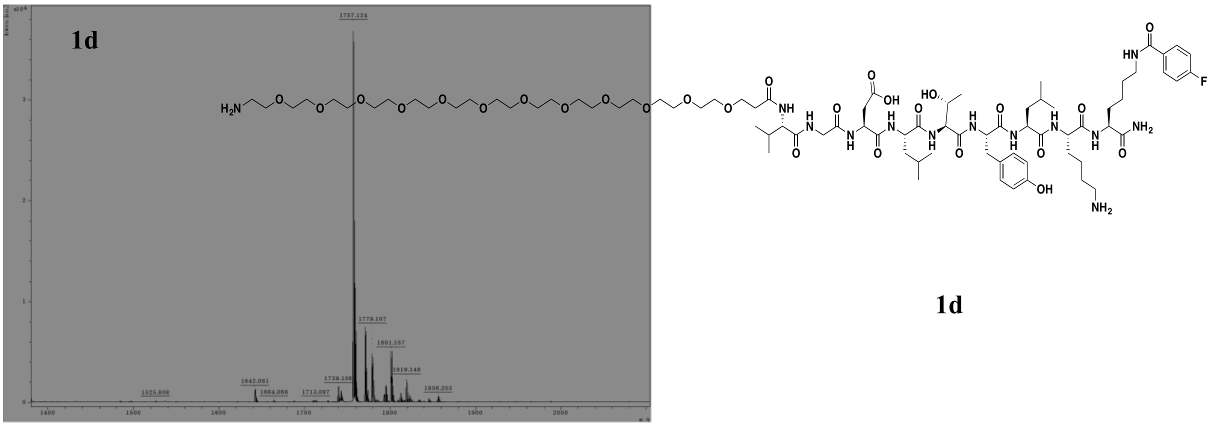

1d

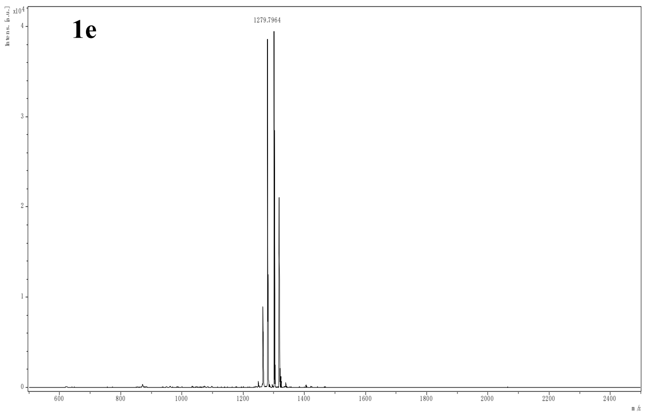

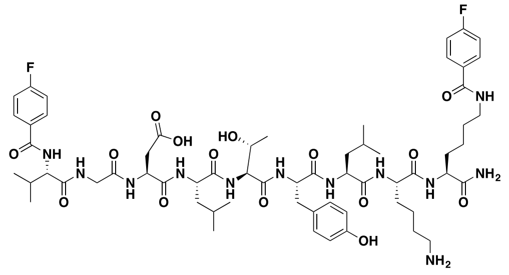

1e

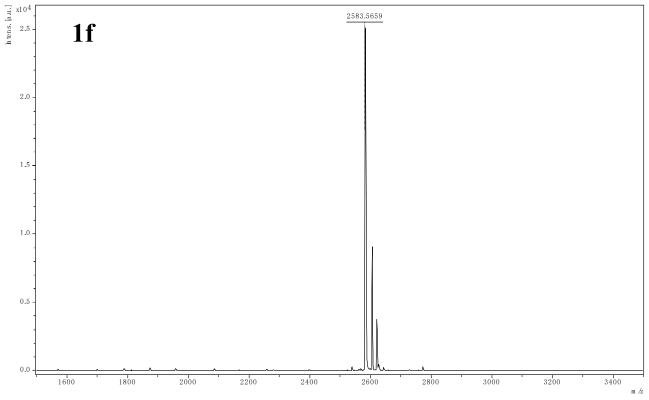

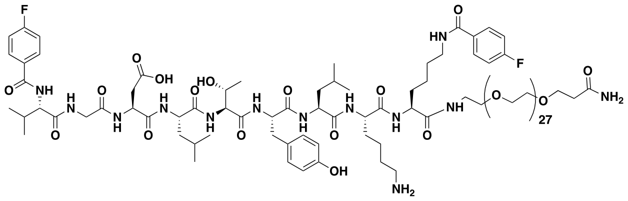

1f

SUPPLEMENTARY FIGURE S2 (CONTINUED)

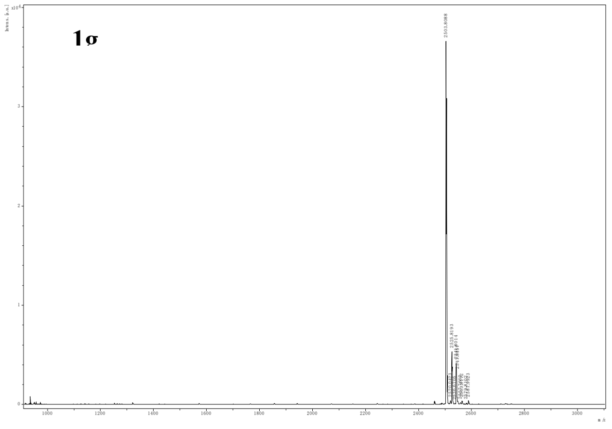

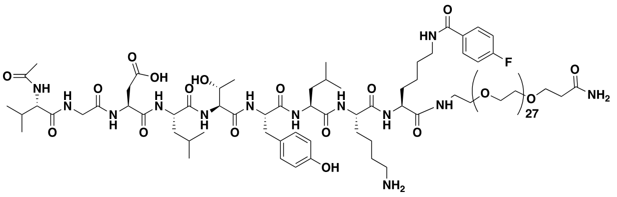

1σ

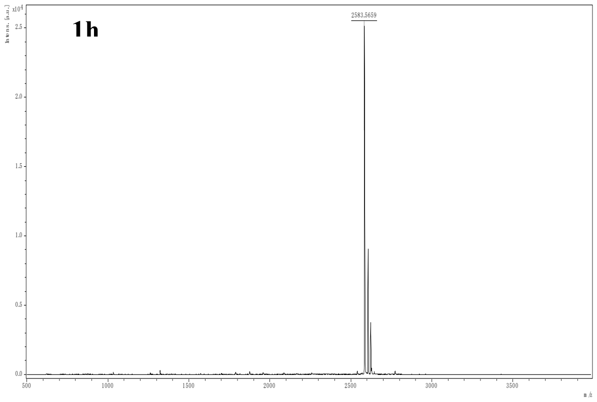

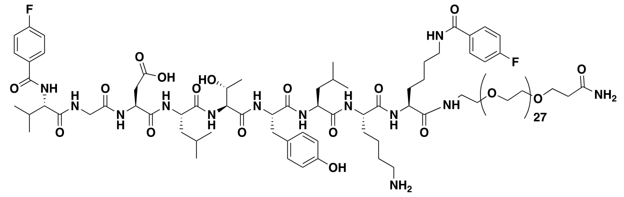

1h

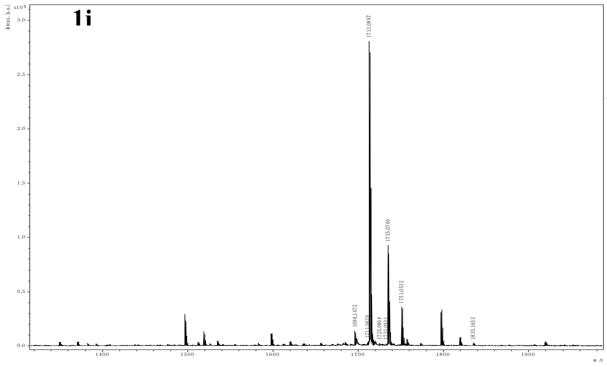

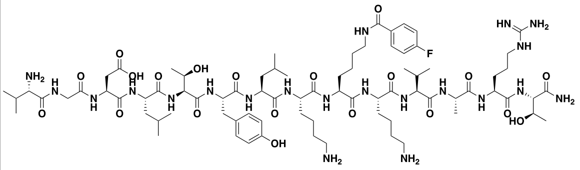

1i

SUPPLEMENTARY TABLE S2. Analytical Radio-HPLC chromatogram confirming the purity of [^{18}F]peptides

ID	Sequence FB =[^{18}F]FB	t_R [min]	RCP [%]	RCY [%]	Molar activity [Ci/µmol]
**1**	VGDLTYLKK(FB)	14.2	>99	5.4 ± 1.2	>1
**2**	RGDLMKLAK(FB)	13.6	78	4	NA
**3**	RGDLADLRK(FB)	12.5	>99	13.4	>1
**5**	RGDLRELAK(FB)	12.7	>99	13.2 ± 10.1	>1
**1a**	FB-VGDLTYLKK	15.7	>99	6.5	>1
**1f**	VGDLTYLKK(FB)-PEG_{28}	16.7	>99	12.8	>1
**1h**	[^{19}F]FB-VGDLTYLKK(FB)-PEG_{28}	19.8	>99	10.9	>1

**1i**	VGDLTYLKK(FB)KVART	12.7	>99	2.8	>1

SUPPLEMENTARY FIGURE S3

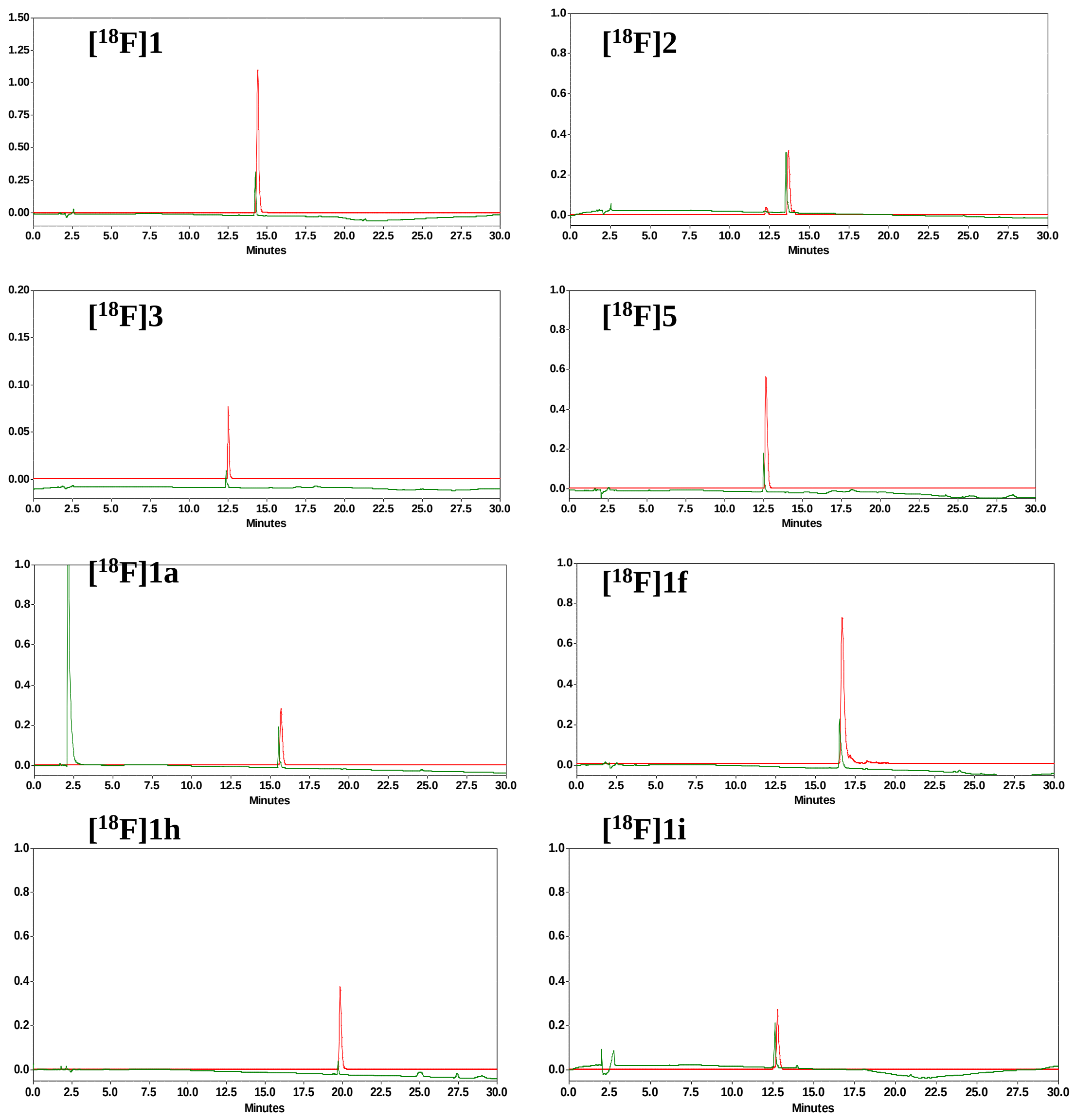

Supplement: Supplementary file 1 [file molecules-24-00309-s001.pdf]
